# Single ventilator for multiple patients during COVID19 surge: matching and balancing patients

**DOI:** 10.1186/s13054-020-03041-y

**Published:** 2020-06-18

**Authors:** Lonnie G. Petersen, James Friend, Sidney Merritt

**Affiliations:** 1 Department of Radiology, School of Medicine, University of California, San Diego, CA USA; 2 Department of Mechanical and Aerospace Engineering, Jacobs School of Engineering, University of California, San Diego, CA USA; 3 Department of Surgery, School of Medicine, University of California, San Diego, CA USA; 4 Department of Anesthesiology, School of Medicine, University of California, San Diego, CA USA

**Keywords:** COVID-19, Patient surge, Mechanical ventilation, Sharing a ventilator

To the editor

With a potential COVID19-induced ventilator shortage, supporting multiple patients on a single ventilator seems a simple solution to maximize resources. Described by Neyman et al. [1], this practice has anecdotally been used in the 2017 Las Vegas mass shooting and more recently in Italy and New York during the COVID-19 pandemic. However, a recent consensus statement from relevant medical associations discouraged the practice based on safety concerns [2]. Beyond cross-contamination and increased dead space, matching patients to ensure appropriate individual ventilation peak pressures (*P*_peak_), tidal volumes (*V*_tidal_), and positive end-expiratory pressures (PEEP) is a concern, especially given the dynamic clinical presentation of the COVID19 patients with complicated acute respiratory distress syndrome (ARDS). The central question remains: *What does it mean to match patients?* How much can they differ before we are no longer saving two lives but risking both?

To illustrate the effect of progressive mismatching, we ventilated two mechanical lungs (TTL3, Michigan Instruments) on a ventilator (840, Puritan Bennett™) using pressure control mode and ARDS-compatible settings (*P*_peak_ = 20–30 cmH_2_O; *R* = 20 bpm; *F*_total_ = 24 l/min; *I* = 1.5 s; PEEP = 8 cmH_2_O) [3]. While keeping patient B at constant pulmonary compliance (0.03 l/cmH_2_O), we let patient A progressively deteriorate in compliance from 0.06 to 0.01 l/cmH_2_O, finally creating a maximum mismatch between patients (see Fig. 1). One-way valves on both inspiratory and expiratory limbs ensured unidirectional flow, which both reduces functional dead space and the risk of cross-contamination between patient A and B, and seemingly also facilitated stable ventilation of B as A deteriorated. Importantly though, simultaneous and opposite changes in compliance made it possible to fatally hypo-ventilate one patient and hyper-ventilate the other without triggering alarms. As ventilator alarms are triggered only by changes in the sum of the pressure/volume of both patients on the circuit, we recommend a narrow alarm range (e.g., *V*_tidal_ ± 200 ml; *F*_total_ ± 1 L; *P*_peak_ ± 5 cmH_2_O). The one-way valves on each expiratory limb prevent backflow but introduce a risk of competing exhalation: a slightly earlier or more forceful expiration from A can (partly) impair B and worsen breath staggering, particularly at higher respiration rates.

**Fig. 1:**
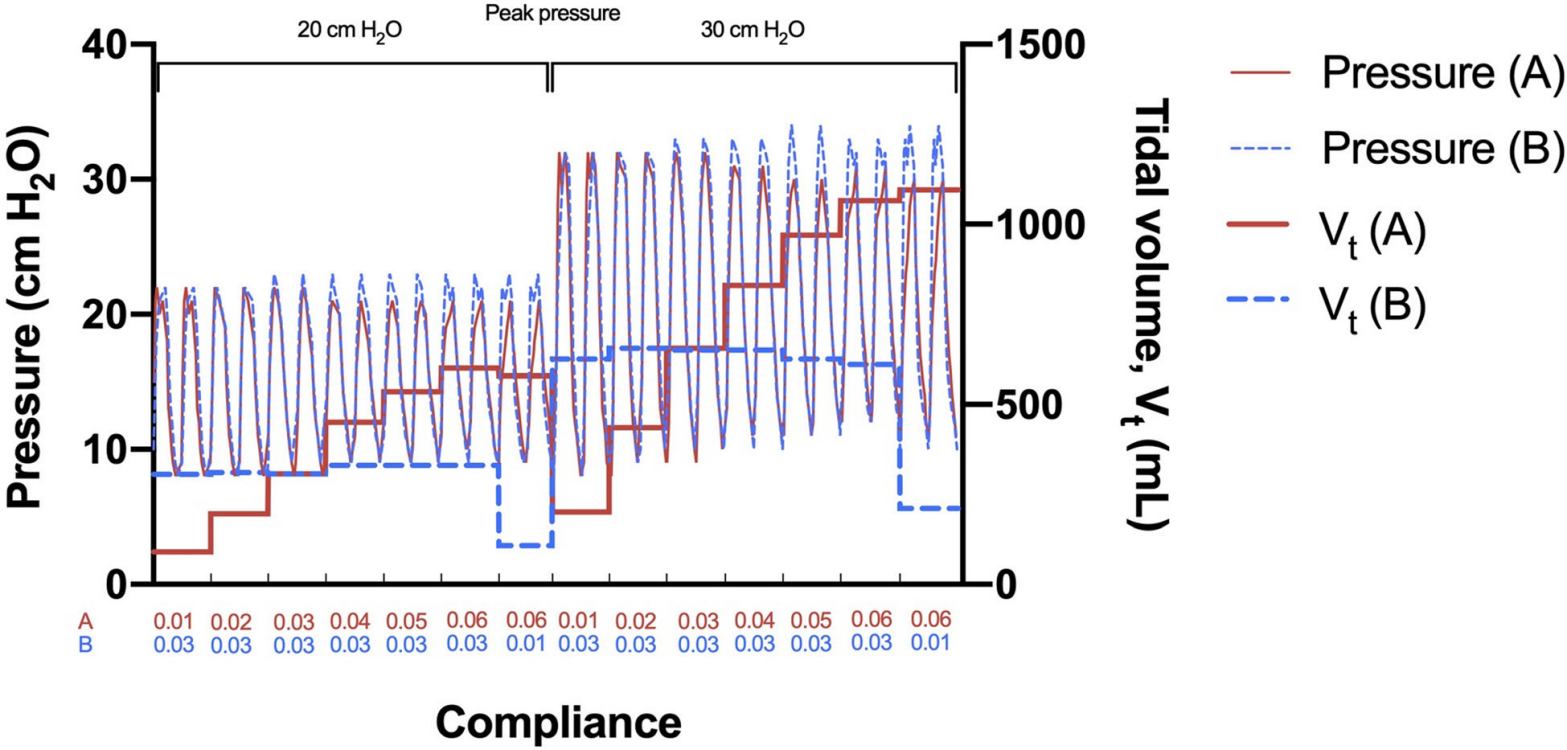
Patients A (red line) and B (blue line) pressure curves and average tidal volumes during positive pressure ventilation, using pressure control mode mechanical ventilation at 20 and 30 cmH_2_O and PEEP of 8 cmH_2_O. One-way valves ensured stable tidal volumes of B while A deteriorated; however, a severe mismatch holds the potential for fatal simultaneous hyper- and hypo-ventilation without triggering alarms

Frequent or constant monitoring of patients and shuffling when a mismatch arises is recommended. Asthma or COPD may increase the rate of fatal mismatch, making the method even more unpredictable. Finally, each class of ventilators requires a specific set up; if the method is considered, use the calm before the patient surge to familiarize, and ameliorate the many risks associated with sharing a ventilator.

## Data Availability

The datasets used and analyzed during the current study are available from the corresponding author on reasonable request.
